# A recent record of *Romanogobioantipai* (Actinopterygii, Cyprinidae, Gobioninae) from the Danube River in Bulgaria

**DOI:** 10.3897/zookeys.825.32434

**Published:** 2019-02-27

**Authors:** Nina G. Bogutskaya, Tihomir Stefanov, Alexander M. Naseka, Oleg A. Diripasko

**Affiliations:** 1 Naturhistorisches Museum Wien, Burgring 7, Vienna 1010, Austria Naturhistorisches Museum Wien Vienna Austria; 2 National Museum of Natural History, 1 Tsar Osvoboditel Blvd, 1000 Sofia, Bulgaria National Museum of Natural History Sofia Bulgaria; 3 Institute of Fisheries and Marine Ecology, 8 Konsulska St, Berdyansk, 71118, Ukraine Institute of Fisheries and Marine Ecology Berdyansk Ukraine

**Keywords:** Danube delta gudgeon, morphology, meristics, distribution, conservation status

## Abstract

The Danube delta gudgeon, *Romanogobioantipai*, has been considered to be extinct because there were no reliable recent observations. The latest record confirmed by a voucher specimen dating from 1992. We report here on a specimen of *R.antipai* collected in 2016 in the Bulgarian sector of the Danube main stream using a bottom drift net at a depth of 8 m. The species determination is supported by morphological examination including discriminant and cluster analyses in comparison with three syntypes and five non-type specimens of *R.antipai*, samples of the *R.kesslerii* species complex and *R.vladykovi*. *Romanogobioantipai* most clearly differs from both *R.kesslerii* and *R.vladykovi* by proportional measurements (caudal peduncle depth, head width, eye horizontal diameter, and interorbital width), from *R.kesslerii* also by the number of scales above and below the lateral line (6 and 4, respectively, (vs. commonly 5 and 3), and from *R.vladykovi*, also by 8½ branched dorsal-fin rays (vs. 7½) and the vertebral caudal region longer than the abdominal vertebral region (abdominal+caudal vertebrae 19+21 or 20+21, vs. commonly 20+20 or variants with a caudal region shorter than the abdominal one). The possibility that *R.antipai* represents a deep-water cophenotype of either *R.kesslerii* or *R.vladykovi*, cannot be excluded. The new record demonstrates that *R.antipai* is still extant in the lower Danube but may be restricted to greater depths in the main channel and the deltaic branches.

## Introduction

*Romanogobio* Bănărescu, 1961 is a genus of bottom-dwelling, rheophilic gudgeons with a wide distribution in temperate Eurasia. Six species were reported from the Danube basin (Kottelat and Freyhof 2007, Friedrich et al. 2018). Among them, *Romanogobioantipai* was described, as *Gobiokessleriantipai*, by Bănărescu (1953: 300, 318) based on a series of syntypes from the Danube delta at Sulina (12 specimens collected by Grigore Antipa before 1909) and the lower reaches of the Danubian tributary Argeş (one specimen collected by Băcescu). In the same paper, representing a study of morphometric features within the “*Gobiokessleri*” group of populations distributed in Romania, Bănărescu also recognized a new form, *Gobiokesslerikessleri* natio *banaticus* (the name is not available from this publication, but available as *Gobiokessleribanaticus* from Bănărescu 1960: 121) and compared both new forms with the nominotypical subspecies. As the correct original spelling of the specific name is *kesslerii* (Kottelat 1997), we use it hereafter.

Bănărescu (1953) distinguished *Romanogobioantipai* from *R.kesslerii* and *R.k.banaticus* on average values of the postorbital distance (10.5% of body length vs 8.5–10%), barbel length (10.5–13% of body length vs 8–11.5%), eye diameter (5.5% of body length vs 5.6–6.4% *R.* excluding *kesslerii* (Dybowsky, 1862) from Bulgaria with 4.8–5.6%, and 75.2% of interorbital distance vs 81.9–98.5%), snout length (9.4% of body length vs 9.4–11.7%), and maximum body depth (18.4% of body length vs 1.8–17.7% excluding *R.kesslerii* from the Dniester with an average of 18.5%). Consequently, no clear differences between the three taxa were presented, and later Bănărescu (1960, 1961) reported the occurrence of specimens morphologically intermediate between *R.antipai* and *R.kesslerii* in the Danube tributaries Ialomiţa, Argeș (Dâmboviţa) and Siret (Bizau and Milcov Rivers), recognized as such also by Bănărescu and Nalbant (1973). Bănărescu (1961, 1999: 151) repeated the most typical features of *R.antipai* from the Danube delta (smaller eye and deeper body), adding smaller body length in adults (“apparently not exceeding 6 cm”), commonly 4 scales (vs commonly 3) between the lateral line and the pelvic-fin origin, caudal-peduncle width at the anal-fin origin commonly about equal to caudal-peduncle depth (vs larger in *kesslerii*), and short lateral blotches (vs commonly elongated in *banaticus*). The distribution range was widened (Bănărescu 1961: 344) to include the lower reaches of the Siret River and its tributaries, the Milcov, Putna, and Birlad Rivers, and the lowest reaches of the Argeş and Ialomiţa Rivers. Similar data were later published in the book on the fishes of Romania (Bănărescu 1964: 454–455, fig. 195).

Bănărescu (1992, 1994a, 1999: 150, fig. 21) again restricted the range of *R.antipai* to the lowest reaches of the Danube, mentioning that before 1959, it was distributed upstream to the Argeş River mouth (some 430 river kilometres). He emphasized that it markedly differed from other members of the *R.kesslerii* complex by its morphological features, which were related to dwelling in deep water of the main stream of the river.

Bănărescu (1992, 1994a, 1999) treated the Danube delta gudgeon as a subspecies (*Gobiokessleriiantipai*) but mentioned that it deserved the rank of species. Kottelat and Freyhof (2007) considered it as a valid species and Friedrich et al. (2018) found no arguments to reject this status.

The sample of *Romanogobioantipai* from the Danube delta at Sulina, described by Bănărescu based on G. Antipa’s collections (before 1909) was not the only one from the Danube delta. Smirnov (1971) provided meristic and morphometric data on a sample of 24 specimens collected by him in April 1961 in the Ukrainian part of the delta (Chilia Arm) near Izmail and identified it as *R.kesslerii*. This sample was later included in the book on Ukrainian fishes by Movchan and Smirnov (1981: 344, tab. 181). Bănărescu (1999: 158) suggested that Smirnov’s (1971) specimens from Izmail belonged to *R.antipai* because they had a deeper body and a smaller eye.

The most recent published record of *R.antipai* from the lower section of the Danube may be that of Marinov (1978) who reported *Gobiokesslerii* from the main course of the Danube in Bulgaria. The true *R.kesslerii* only occurs in middle reaches of tributaries and has not been recorded from the deep main stream of the Danube (e.g., Chichkoff 1937, Mihailova 1970, Dikov et al. 1994).

The absence of recent records of *R.antipai* lead some authors to the conclusion that the species might be extinct (Bănărescu 1994b, Kottelat 1997). Its conservation status was later evaluated as extinct (Kottelat and Freyhof 2007, Freyhof and Kottelat 2008) because it was supposed that all known ichthyological surveys conducted since the 1960s to 2003 in suitable habitats in the Danube delta had failed to find it; however, this statement is not entirely correct as there is a specimen in Natural History Museum ‘Grigore Antipa’ collected in 1992 (described below).

**Figure 1.**
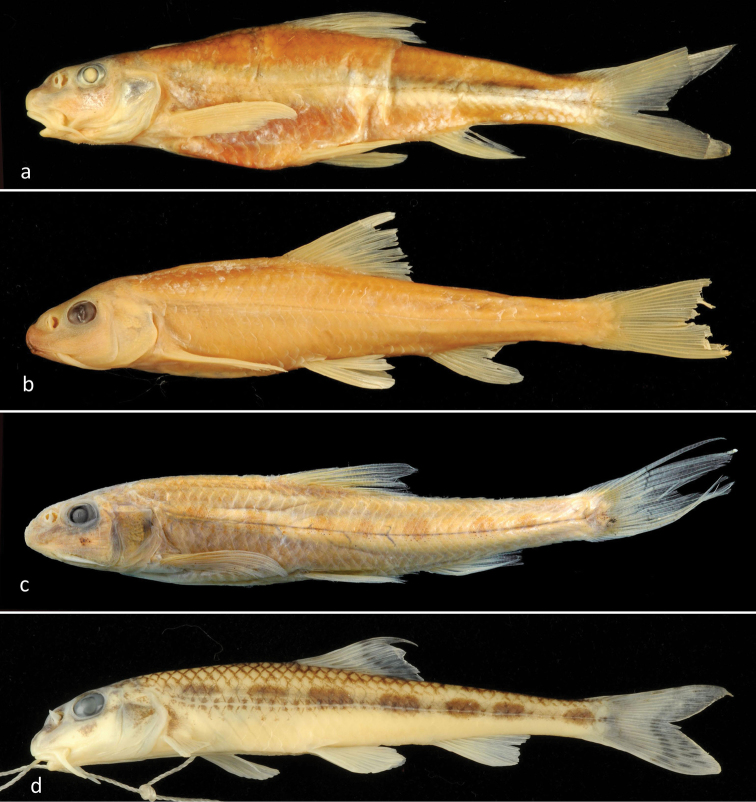
Lateral view of *Romanogobioantipai*NMNHS specimen 68.7 mm SL, Danube at Vetren (**a**) MGAB/BN760 specimen 64.9 mm SL, lower Argeş R. (**b**) ANSP syntype 47.8 mm SL, Sulina (**c**) and *R.kesslerii*NMNHS, 65.7 mm SL, Tsibritsa River (**d**).

**Figure 2.**
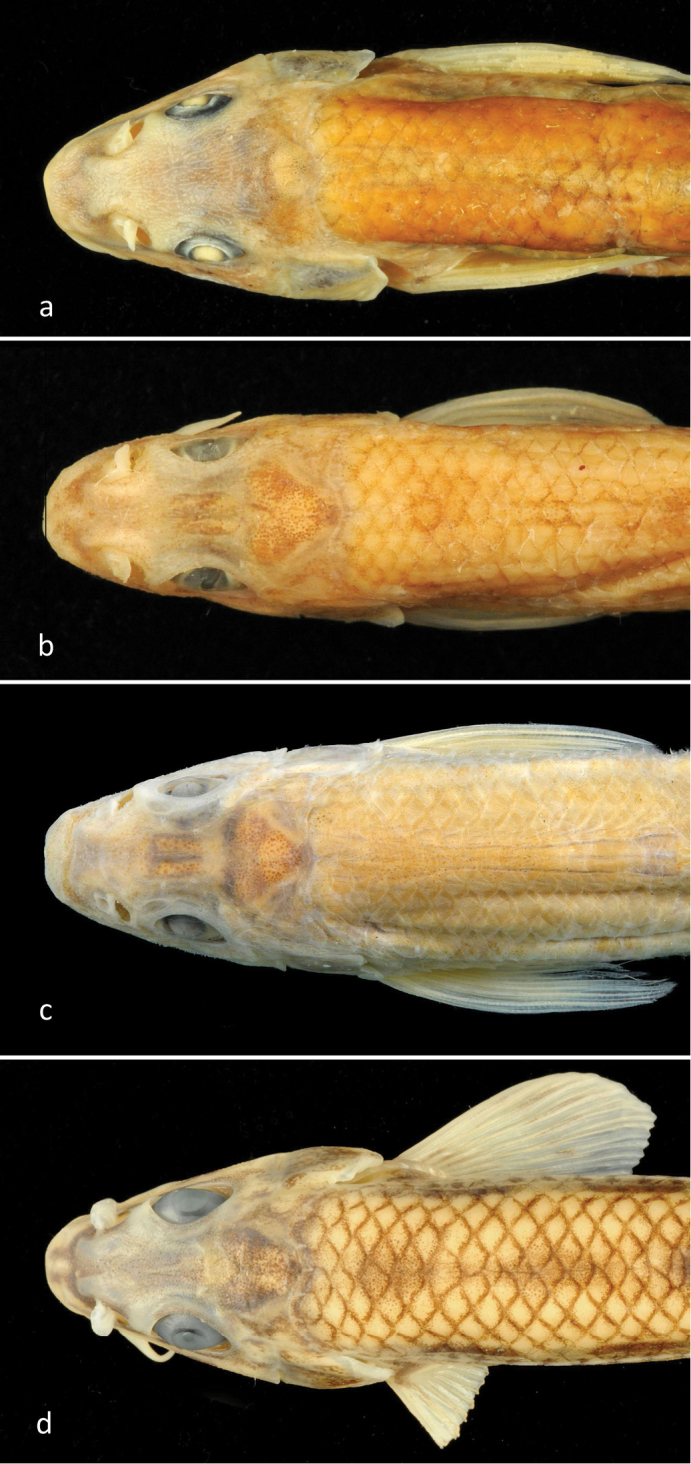
Dorsal view of the head of *Romanogobioantipai*, NMNHS specimen 68.7 mm SL, Danube at Vetren (**a**) MGAB/BN759, SL 47.7 mm, Sulina (**b**) ANSP syntype 47.8 mm SL, Sulina (**c**) and *R.kesslerii*NMNHS, 65.7 mm SL, Tsibritsa River (**d**).

In 2016, TS collected a gudgeon specimen in the Bulgarian sector of the Danube main stream using a bottom drift net at a depth of 8 m, near the village of Vetren (river kilometer 395). This specimen (Figs 1–2) was preliminarily identified by AN as *Romanogobioantipai* because of its small eye and 8½ branched dorsal-fin rays distinguishing it from both *R.kesslerii* and *R.vladykovi* (Fang, 1943). The present note is devoted to a comparative description of the specimen to test this hypothesis.

## Material and methods

Methods for counting fin rays and scales, and for measurements, follow Kottelat and Freyhof (2007) except that head length, postorbital length, and interorbital width include the skin fold. In the examined samples, standard length is used for some relative measurements. Body length, which is the length to the posterior margin of the posterior-most scale on the base of the caudal fin (called standard length II by Holčík et al. (1989: fig. 12), is also measured and the data are compared for diagnostic characters taken from the literature. All measurements were made point-to-point with a dial caliper and recorded to the nearest 0.1 mm. Vertebral counts taken from radiographs follow the scheme by Naseka (1996). To avoid probable discrepancy in lateral-line count, we provide not only the number of lateral-line scales to the posterior margin of the hypurals but also numbers of total lateral scales and total lateral-line scales. Statistical analyses were done using Microsoft Excel, Statistica 6.0 (Statistic for Windows. Statsoft; Discriminant Functional Analysis, DFA), and SPSS Statistics V23.0 (IBM SPSS; Cluster Analysis, CA).

### Abbreviations

ANSP, Academy of Natural Sciences, Philadelphia, USA; NB, Bănărescu Nalbant Ichthyology Collection (now in Muzeul de Istorie Naturala ‘Grigore Antipa’); ICBB, Institutui of Stiinte Biologice, Bucharest, Romania; IUCN, International Union for Conservation of Nature; MGAB, Muzeul de Istorie Naturala ‘Grigore Antipa’, Bucharest, Romania; NMNHS, National Museum of Natural History, Sofia, Bulgaria; NMW, Naturhistorisches Museum Wien, Vienna, Austria. BL, body length; HL, lateral head length; rkm, river kilometer; SD, standard deviation; SL, standard length.

### Material examined

We specifically selected for comparison mostly those specimens of *R.kesslerii* that were donated and/or identified by Petru Bănărescu and followed his original descrimination of the forms within the *R.kesslerii* species complex. Specimens of *Romanogobiovladykovi* were selected from localities geographically close to Bulgaria and of a comparable length range.

NMNHS [no number], SL 68.7 mm, Bulgaria: Danube near Vetren, 395 rkm, 44.142637N, 27.029662E, 8 July 2016, coll. T. Stefanov.

*Romanogobioantipai*. All from Romania. Type material: MGAB 49908 (as *Gobiokessleriantipai*), 1, SL 46.2 mm, labelled as holotype, Romania: [Danube at] Sulina, before 1909, leg. G. Antipa; MGAB (ISBB 0519), 1, SL 50.8 mm, labelled as paratype, same data as MGAB 49908; ANSP 98961 (as *Gobiokessleriantipai*), 1, SL ca. 47.8 mm, labelled as paralectotype (misspelled as lectoparatype), same data as MGAB 49908, don. P. Bănărescu as paratype; non-type: MGAB (ISBB 0714, BN760, as *Gobiokessleriantipai*), 3, SL 64.9, 28.6 and 26.2 mm, Romania: lower Argeş River at Olteniţa, 26 July 1961, coll. and det. P. Bănărescu; MGAB (BN759, as *Gobiokessleriantipai*), 1, SL 47.7 mm, Romania: Sulina Branch, ”27–28^th^ miles”, Danube delta, Oct. 1992; MGAB (ISBB 3567, BN758, as *Gobiokessleriantipai*), 1, SL 30.1 mm, Romania: Saint Gheorghe Branch, Danube delta, no date, coll. V. Leonte. ANSP 98961 was examined on photos (lateral, dorsal, and ventral aspects) and an X-radiograph.

*Romanogobiobanaticus* (but see Friedrich et al. 2018: 346 on *R.carpathorossicus* (Vladykov, 1931) as senior synonym of this species). NMW 65539, 30, SL 31.1–7.9 mm, Romania: Timiş at Urseni, Timişoara, 6 Sept. 1962, don. and det. P. Bănărescu as *Gobiokessleriibanaticus*.

*Romanogobiokesslerii* s.l.. NMW65532, 12, SL 33.3–57.9 mm, Romania: Tur at Turulung, northeast of Satu-Mare, 5 Sept. 1963, don. and det. P. Bănărescu, as intermediate between *Gobiokessleriikesslerii* and *Gobiokessleriibanaticus*; NMW 65538, 9, SL 39.4–74.7 mm; Romania: Milcov at Focsani, Moldau, 14 Sept. 1963, don. and det. P. Bănărescu as *Gobiokessleriantipai* with a comment: “not very typical”, as *Gobiokesslerikessleri* in Bănărescu (1999: 146); NMNHS [no number], 10, SL 55.1–66.5 mm; Bulgaria: Tsibritsa River [right tributary to Danube, NW Bulgaria] near Yakimovo, 43.62245N, 23.33022E, 18 July 2012, coll. T. Stefanov.

*Romanogobiokessleriikesslerii*. NMW 60250, 6, SL 50.1–61.2 mm; Romania: Areş River at mouth of Mureş, Transsylvania, 2 Oct.1949, don. and det. P. Bănărescu (in *Gobio*); NMW 65535, 4, SL 50.6–61.5 mm; Romania: Bereteu at Roşiori-Bihor, north of Oradea, 4 Sept. 1963, don. and det. P. Bănărescu (in *Gobio*); NMW 90883, 2, SL 85.3–91.2 mm; Ukraine: Smotrich River at Kamenetz Podol’ski [Dniester drainage], 7 May 1921, det. P. Bănărescu, 1991 (in *Gobio*).

*Romanogobiovladykovi*. NMW 53356, 2, SL 76.7–81.4 mm; Serbia: Nisch [Niš, Great Morava-Danube], Dec. 1894; NMW65537, 20, SL 46.4–81.4 mm; Romania: Timiš River at Peciu Nou [Danube drainage], 28 Aug. 1863; NMW 60234, 2, SL 73.2–77.2 mm; Romania: Bega R. [Danube drainage], Banat, Sept. 1943.

## Results

General appearence of the NMNHS presumed *R.antipai* specimen from the Danube at Vetren is shown in Figs 1–2 together with a syntype and a non-type *R.antipai* specimen in comparison to *R.kesslerii* and *R.vladykovi*. Counts, descriptive states of the pectoral-fin length, and measurements are presented in Tables 1, 2. Examined character states in this specimen coincide considerably with those in the three type specimens of *R.antipai* and five non-type specimens, and demonstrate its differences from the samples of both the *R.kesslerii* species complex and *R.vladykovi*. As the standard length averages 96.8% of the BL (calculated in the *Romanogobio* material examined in this study), the difference between relative measurements (in % SL and in % BL) is slight and the morphometric character states that have been considered as diagnostic for *R.antipai* vs. *R.kesslerii* are confirmed. They include maximum body depth, 19–25% SL (17–25.5% of BL; in parentheses, data from Bănărescu (1953, 1961, 1999) and Movchan and Smirnov (1981) are summarized); caudal peduncle depth, 8–9% SL (7–9% of BL) and 35–38.5% of caudal peduncle length; eye diameter, 5–6% SL (5–6% of BL), 20–23% HL (18–24% HL), and 59–68.5% of interorbital width (61–81% of interorbital width). The NMNHS specimen has 6 and 4 scales, respectively, above (to the dorsal-fin origin) and below (to the pelvic-fin origin) the lateral line similar to the three type specimens of *R.antipai* and the topotypical specimen from Sulina thus confirming the opinion (Kottelat and Freyhof 2007, Friedrich et al. 2018) that this character is one of the most dependable diagnostic characters for the species. All other counts are identical or very close in the NMNHS specimen and *R.antipai* examined in this study (Table 1). None of the *R.antipai* specimens had 7½ branched dorsal-fin rays thus confirming its main difference from *R.vladykovi* characterized by 7½ branched dorsal-fin rays (Naseka et al. 1999; Naseka 2001).

**Table 1.** Meristic data for examined specimens of *Romanogobioantipai* and *R.kesslerii* species complex.

	Dorsal-fin branched rays			Anal-fin branched rays		Pectoral fin relative to pelvic-fin origin				Scales between lateral line and dorsal-fin origin		Scales between lateral line and pelvic-fin origin		
	7½	8½	9½	6½	7½	not reaching	almost reaching	reaching	behind	5	6	2	3	4
*R.antipai*MGAB 49908 syntype		1		1		1					1			1
*R.antipai*MGAB/ISBB 0519 syntype		1		1		1					1			1
*R.antipai*ANSP 98961 syntype		1		1		1					1			1
Non-type MGAB*R.antipai*, n=5		5		1		4	1				5			5
Presumed *R.antipai*NMNHS specimen		1		1			1				1			1
*R.k.kesslerii*. NMW 60250, 65535. Danube drainage, n=10		10		10		7	1	2		10			10	
*R.k.kesslerii*. NMW 908803, Dniester drainage, n=2		2		2		2				2			2	
*R.banaticus*. NMW 65539, Danube drainage, n=30		27	3	30		26	3	1		29	1		30	
*R.kesslerii* s.l. NMW 65532, NW Romania, n=12		12		12		6	1	5		12		1	11	
*R.kesslerii* s.l. NMW 65538, NE Romania, n=9		8	1	6	3		5	3	1	9			9	
*R.kesslerii* s.l. NMNHS, NW Bulgaria, n=10		10		10	10	6	2	1	1	10			10	
*R.vladykovi*NMW 53356, 60234, 65537, Danube drainage, n=24	24			24		24				10	14		15	9

**Table 1.** Continued.

	Predorsal abdominal vertebrae		Abdominal vertebrae				Pre-anal caudal vertebrae			Caudal vertebrae			Total vertebrae				
	10	11	18	19	20	21	2	3	4	19	20	21	38	39	40	41	42
*R.antipai*ANSP 98961 syntype	1			1				1			1			1			
Non-type MGAB*R.antipai*, n=5	2	3		4	1			3	2			5			4	1	
Presumed *R.antipai*NMNHS, n=1		1			1			1				1				1	
*R.k.kesslerii*NMW 60250, 65535. Danube drainage, n=10	2	8		1	8	1	7	3		3	5	2		2	7	1	
*R.k.kesslerii*NMW 908803, Dniester drainage, n=2		2			2			2			2				2		
*R.banaticus*. NMW 65539, Danube drainage, n=30	9	21		26	4		4	21	5	2	19	8	1	18	10	2	
*R.kesslerii* s.l. NMW 65532, NW Romania, n=12	1	11		7	5		5	7		1	7	4		4	8		
*R.kesslerii* s.l. NMW 65538, NE Romania, n=9	1	8		3	6		4	5		1	5	3		1	8		
*R.kesslerii* s.l. NMNHS, NW Bulgaria, n=10	10		2	8			2	8		2	8		4	6			
*R.vladykovi* Danube drainage, Romania and Ukraine, n=46 (from Naseka 2001).	9	37		4	38	4	12	46	8	4	29	12		3	31	11	1
*R.vladykovi*NMW 53356, 60234, 65537, Danube drainage, Romania and Serbia, n=24	3	21			19	5	12	12		5	15	4		2	16	6	

**Table 1.** Continued.

	Total lateral-series scales						Total lateral-line scales					Lateral-line scales to posterior hypural margin					
	39	40	41	42	43	44	39	40	41	42	43	37	38	39	40	41	42
*R.antipai*MGAB 49908 syntype				1					1					1			
*R.antipai*MGAB/ISBB 0519 syntype					1					1					1		
*R.antipai*ANSP 98961 syntype			1						1					1			
Non-type MGAB*R.antipai*, n=5			1		3	1			2	3	1		1	2	1		1
Presumed *R.antipai*NMNHS, n=1						1					1						1
*R.k.kesslerii*NMW 60250, 65535. Danube drainage, n=10			3	4	3			1	2	5	2		2	2	5	1	
*R.k.kesslerii*NMW 908803, Dniester drainage, n=2			1	1					1	1				1	1		
*R.banaticus*NMW 65539, Danube drainage, n=30			8	9	3			1	8	9	2		4	6	9	1	
*R.kesslerii* s.l. NMW 65532, NW Romania, n=12		2	4	6				4	3	5		2	2	6	2		
*R.kesslerii* s.l. NMW 65538, NE Romania, n=9			7	2				3	4	2			3	4	2		
*R.kesslerii* s.l. NMNHS, NW Bulgaria, n=10		6	3	1			2	5	2	1			6	4			
*R.vladykovi*NMW 53356, 60234, 65537, Danube drainage, Romania and Serbia, n=24	1	7	5	10	1		1	8	6	8	1	1	9	8	6		

**Table 2.** Measurements and counts for examined specimens of *R.antipai*, *R.kesslerii* and *R.vladykovi*. Gap between ranges or ranges only slightly overlapping: * between *R.antipai* and *R.kesslerii*, ** between *R.antipai* and both *R.kesslerii* and *R.vladykovi*, *** between *R.antipai* and *R.vladykovi*; **** no statistical difference; remaining characters display statistically significant differences (Kruskal-Wallis test, p<0.01) but ranges overlapping.

Characters	*R.antipai* n=6					*R.kesslerii* n=26				*R.vladykovi* n=22			
	* R. antipai * NMNHS	min	max	Mean	SD	min	max	Mean	SD	min	max	Mean	SD
SL, mm	68.67	46.20	68.67	54.44	9.743	45.11	74.69	59.53	5.776	46.39	81.42	60.21	10.310
Body depth at dorsal-fin origin (% SL)*	25.43	18.56	25.43	20.37	2.671	15.59	18.63	16.88	0.834	17.50	23.00	19.20	1.277
Depth of caudal peduncle (% SL)*	9.20	8.13	9.20	8.49	0.388	6.33	7.67	7.04	0.358	8.30	10.15	9.06	0.416
Depth of caudal peduncle (% length of caudal peduncle)**	38.47	34.80	38.47	36.72	1.416	24.90	33.44	30.23	2.599	39.32	49.69	44.12	3.117
Predorsal length (% SL)	46.56	44.43	46.56	45.57	0.832	43.41	49.32	46.73	1.415	46.29	49.40	47.57	0.949
Postdorsal length (% SL)	41.90	37.29	41.90	39.89	1.945	39.56	47.49	43.91	1.884	39.48	43.83	41.56	1.432
Prepelvic length (% SL)	48.89	47.76	50.60	48.79	0.989	44.79	49.78	47.45	1.218	48.41	52.79	50.65	1.423
Preanal length (% SL)	70.21	69.60	72.20	70.74	0.875	67.12	73.01	69.91	1.653	69.90	76.28	73.23	1.181
Pectoral – pelvic-fin origin length (% SL)	23.92	22.95	24.60	23.88	0.650	21.15	25.42	23.51	0.865	23.29	27.32	25.58	1.423
Pelvic – anal-fin origin length (% SL)	23.13	21.70	23.13	22.17	0.535	18.66	24.84	21.55	1.374	20.12	24.59	22.86	1.080
Caudal peduncle length (% SL)	23.93	22.61	24.00	23.15	0.632	20.21	29.11	23.80	2.502	18.68	22.98	20.63	1.440
Pectoral-fin length (% SL)	23.18	22.05	23.18	22.55	0.440	19.69	24.00	22.20	1.320	18.49	22.89	20.75	1.162
Pelvic-fin length (% SL)	19.67	17.52	19.67	18.54	0.732	16.13	19.10	17.94	0.743	14.22	18.69	16.35	1.266
Head length (% SL)****	25.99	24.80	25.99	25.34	0.499	23.35	27.20	25.75	0.986	24.05	27.60	26.21	0.794
Head length (% body depth)	102.23	102.23	132.63	121.08	10.537	127.58	186.49	153.40	11.409	111.64	156.60	137.16	11.096
Head depth at nape (% SL)*	17.21	14.90	17.21	16.10	0.791	13.06	15.84	14.38	0.734	14.60	16.57	15.79	0.531
Head depth at nape (% HL)	66.22	59.30	68.70	64.48	3.209	52.81	59.43	55.82	1.971	57.09	64.32	60.27	1.975
Barbel length (% HL)	34.29	34.29	47.90	41.41	5.128	26.90	50.00	39.45	5.569	25.42	40.21	31.97	4.092
Maximum head width (% SL)**	15.42	14.61	16.20	15.38	0.653	13.13	15.19	13.83	0.547	12.90	15.77	14.70	0.767
Maximum head width (% HL)	59.33	58.20	64.00	61.25	2.719	50.03	56.98	53.64	2.192	49.83	59.08	55.98	2.024
Snout length (% SL)****	11.33	8.40	11.33	9.75	0.997	8.94	11.98	10.57	0.625	9.07	11.74	10.16	0.696
Snout length (% HL)	43.59	36.00	43.60	39.40	2.654	37.86	46.38	41.04	2.337	35.93	42.79	38.75	1.903
Eye horizontal diameter (% SL)**	5.24	5.20	5.94	5.49	0.285	5.78	8.15	6.63	0.642	6.36	8.28	7.48	0.463
Eye horizontal diameter (% HL)***	20.17	20.17	23.97	22.06	1.301	22.72	29.97	26.25	1.809	24.69	32.29	28.54	1.874
Eye horizontal diameter (% interorbital width)**	58.73	58.73	67.50	63.35	4.101	75.25	130.37	103.18	14.774	86.33	121.43	103.22	7.479
Postorbital distance (% HL)****	44.15	43.20	48.76	45.89	2.111	37.27	48.97	42.38	2.756	37.93	45.81	41.91	2.408
Interorbital width (% SL)*	8.93	8.15	8.93	8.56	0.375	5.23	8.33	6.56	0.811	6.74	7.96	7.26	0.368
Interorbital width (% HL)**	34.34	32.48	35.78	34.25	1.316	19.46	31.26	25.45	2.970	25.12	30.25	27.70	1.384
Number of predorsal vertebrae****	11	10.00	11.00	10.67	0.516	10.00	11.00	10.50	0.510	10.00	11.00	10.82	0.395
Number of abdominal vertebrae***	19	19.00	19.00	19.00	0.000	18.00	21.00	19.42	0.703	20.00	21.00	20.18	0.395
Number of caudal vertebrae	21	20.00	21.00	20.83	0.408	19.00	21.00	20.00	0.632	19.00	21.00	19.91	0.610
Number of preanal caudal vertebrae	4	3.00	4.00	3.50	0.548	2.00	3.00	2.58	0.504	2.00	3.00	2.45	0.510
Total vertebrae****	40	39.00	40.00	39.83	0.408	38.00	41.00	39.42	0.809	39.00	41.00	40.09	0.610
Difference between abdominal and caudal numbers***	-2	-2.00	-1.00	-1.83	0.408	-2.00	2.00	-0.58	1.065	-1.00	2.00	0.27	0.827
Dorsal-fin branched rays (without ½)***	8	8.00	8.00	8.00	0.000	8.00	8.00	8.00	0.000	7.00	7.00	7.00	0.000
Scales in lateral row	44	41.00	44.00	42.67	1.033	40.00	42.00	41.08	0.744	39.00	43.00	41.14	1.037
Lateral-line scales (total)***	43	41.00	43.00	42.00	0.894	39.00	42.00	40.73	1.002	39.00	43.00	41.00	1.024
Lateral-line scales (to posterior margin of hypurals)***	42	39.00	42.00	40.33	1.366	38.00	40.00	38.88	0.766	37.00	40.00	38.77	0.869
Scales above lateral line*	6	6.00	6.00	6.00	0.000	4.00	5.00	4.96	0.196	5.00	6.00	5.59	0.503
Scales below lateral line*	4	4.00	4.00	4.00	0.000	3.00	3.00	3.00	0.000	3.00	4.00	3.32	0.477

### Note on syntypes of *R.antipai*

As already clarified (Kottelat 1997), the species group name *antipai* is based on 13 syntypes (Bănărescu 1953: 300) without any catalogue numbers. Soon after, Bănărescu (1961: 344) designated a holotype (“Mus. Gr. Antipa Bukarest, Col. Ichth. Nr. 4) but this action is not valid (Art. 74.5 of the International Code of Zoological Nomenclature; International Commission on Zoological Nomenclature 1999). The referred article of the Code says that a subsequent use of the term “holotype” does not constitute a valid lectotype designation *unless* (italics ours) the author, when wrongly using that term, explicitly indicated that he or she was selecting from the type series that particular specimen to serve as the name-bearing type. We do not know a publication by Petru Bănărescu where he used the term holotype for that specimen *explicitly* indicating its name-bearing role. However, it cannot be excluded that a valid lectotype designation has been already undertaken by someone because the ANSP syntype is labelled as a paralectotype.

## Comparisons

The three examined samples of *Romanogobiokesslerii* s.l. demonstrate a statistically significant difference in ten morphometric and five meristic characters (Table 3) but the ranges of character values overlap considerably and the number of specimens is small. We combined all specimens in a single sample in order to estimate general ranges of character values without a special analysis of variation within the *R.kesslerii* complex.

**Table 3.** Measurements and counts for examined specimens of *Romanogobiokesslerii* species complex. * refers to characters demonstrating statistically significant differences (Kruskal-Wallis test, p<0.01).

Characters	*R.kesslerii* Danube n=10				*R.kesslerii* Romania n=6				*R.kesslerii* Bulgaria n=10			
	min	max	Mean	SD	min	max	Mean	SD	min	max	Mean	SD
SL, mm	50.10	63.42	59.06	3.952	45.11	74.69	57.37	10.157	55.06	66.51	61.31	3.584
Body depth at dorsal-fin origin (% SL)	15.59	17.93	16.81	0.916	16.54	18.30	17.39	0.649	15.73	18.63	16.65	0.787
Depth of caudal peduncle (% SL)*	7.08	7.67	7.37	0.195	6.79	7.34	7.04	0.211	6.33	7.08	6.70	0.214
Depth of caudal peduncle (% length of caudal peduncle)	24.90	33.24	28.84	3.215	28.24	32.85	31.17	1.629	27.17	33.44	31.05	1.865
Predorsal length (% SL)	46.10	48.81	47.39	0.874	44.23	46.70	45.90	0.934	43.41	49.32	46.56	1.826
Postdorsal length (% SL)*	44.25	47.49	45.58	1.260	41.26	44.25	43.27	1.133	39.56	44.64	42.63	1.537
Prepelvic length (% SL)	46.33	49.78	47.84	1.324	46.27	48.69	47.32	0.976	44.79	49.33	47.15	1.250
Preanal length (% SL)	67.12	73.01	70.44	2.227	68.81	71.04	69.90	0.842	67.14	71.01	69.39	1.274
Pectoral – pelvic-fin origin length (% SL)	22.95	23.89	23.27	0.335	23.18	25.18	23.92	0.705	21.15	25.42	23.51	1.230
Pelvic – anal-fin origin length (% SL)	18.66	22.29	21.06	1.307	20.72	22.16	21.34	0.467	18.91	24.84	22.16	1.644
Caudal peduncle length (% SL)*	24.49	29.11	26.35	1.642	21.15	24.14	23.18	1.139	20.21	23.70	21.62	1.099
Pectoral-fin length (% SL)*	22.82	24.00	23.24	0.362	20.43	23.99	22.47	1.167	19.69	23.09	21.01	1.063
Pelvic-fin length (% SL)	17.69	19.10	18.36	0.451	17.19	18.71	18.09	0.508	16.13	18.74	17.44	0.832
Head length (% SL)	25.54	27.20	26.40	0.458	23.35	26.66	25.47	1.181	24.25	26.86	25.28	0.977
Head length (% body depth)	144.23	186.49	158.35	12.847	127.58	159.58	147.35	11.663	139.47	170.77	152.09	8.209
Head depth at nape (% SL)*	13.97	15.84	14.91	0.697	13.83	14.97	14.49	0.410	13.06	14.56	13.79	0.454
Head depth at nape (% HL)	53.04	59.43	56.36	2.349	54.72	59.23	57.01	1.428	52.81	56.20	54.56	1.058
Barbel length (% HL)	32.95	47.64	39.49	5.449	37.63	50.00	43.50	4.540	26.90	43.61	36.99	5.233
Maximum head width (% SL)	13.18	14.30	13.61	0.404	13.13	15.19	14.08	0.657	13.21	14.73	13.91	0.571
Maximum head width (% HL)*	50.03	52.57	51.25	0.976	53.24	56.98	55.35	1.504	53.15	56.43	55.01	0.861
Snout length (% SL)*	10.37	11.20	10.69	0.264	8.94	10.41	9.85	0.534	10.21	11.98	10.89	0.616
Snout length (% HL)*	39.37	42.13	40.40	0.869	37.86	39.61	38.66	0.687	38.32	46.38	43.12	2.260
Eye horizontal diameter (% SL)	6.20	8.15	7.03	0.772	5.78	7.26	6.47	0.570	5.92	6.77	6.31	0.244
Eye horizontal diameter (% HL)	25.14	29.97	27.47	1.896	25.31	27.57	26.29	0.802	22.72	27.36	25.00	1.321
Eye horizontal diameter (% interorbital width)	78.47	130.37	103.10	18.289	75.25	112.80	93.59	14.198	97.90	122.36	109.01	7.799
Postorbital distance (% HL)	39.71	48.97	43.11	3.323	42.00	45.47	43.86	1.350	37.27	43.56	40.75	2.001
Interorbital width (% SL)*	6.04	7.90	7.00	0.635	6.39	8.33	7.06	0.709	5.23	6.24	5.81	0.367
Interorbital width (% HL)*	22.93	30.92	26.52	2.674	25.06	31.26	27.71	2.241	19.46	25.43	23.03	1.827
Number of predorsal vertebrae*	10	11	10.80	0.422	10	11	10.83	0.408	10	10	10.00	0.000
Number of abdominal vertebrae*	19	21	20.00	0.471	19	20	19.50	0.548	18	19	18.80	0.422
Number of caudal vertebrae	19	21	19.90	0.738	20	21	20.50	0.548	19	20	19.80	0.422
Number of preanal caudal vertebrae	2	3	2.30	0.483	2	3	2.67	0.516	2	3	2.80	0.422
Total vertebrae*	39	41	39.90	0.568	40	40	40.00	0.000	38	39	38.60	0.516
Difference between abdominal and caudal numbers	-2	2	0.10	1.101	-2	0	-1.00	1.095	-2	0	-1.00	0.667
Dorsal-fin branched rays (without ½)	8	8	8.00	0.000	8	8	8.00	0.000	8	8	8.00	0.000
Scales in lateral row*	41	42	41.50	0.527	41	42	41.33	0.516	40	42	40.50	0.707
Lateral-line scales (total)	40	42	41.10	0.994	40	42	41.00	0.894	39	42	40.20	0.919
Lateral-line scales (to posterior margin of hypurals)*	39	40	39.50	0.527	38	40	38.67	0.816	38	39	38.40	0.516
Scales above lateral line	4	5	4.90	0.316	5.0	5	5.00	5.0	5	5	5.00	0.000
Scales below lateral line	3	3	3.00	0.000	3.0	3	3.00	3.0	3	3	3.00	0.000

As can be seen from Table 2, examined specimens of *R.antipai* including the NMNHS specimen from the Danube at Vetren, most clearly (with a gap or ranges only slightly overlapping) differ from both *R.kesslerii* and *R.vladykovi* by the caudal peduncle depth (35–38.5% caudal peduncle length vs 25–33 and 39–50, respectively), a wider head (58–64% HL vs 50–59), a smaller eye (5–6% SL and 59–67.5% interorbital width vs 6–8 and 75–130), and a wider interorbital space (32.5–36% HL vs 19.5–31; 8–9% SL vs 5–8) with shallow orbital notches (Fig. 2a–c). *Romanogobioantipai* can be further distinguished from *R.kesslerii*, besides the number of scales above and below the lateral line (6 and 4, respectively, in all examined *R.antipai* vs commonly 5 and 3 in *R.kesslerii* s.l.), by a deeper body (19–25% SL vs 16–19), a deeper caudal peduncle (8–9% SL vs 6–8), and a deeper head (59–69% HL vs 53–59).

Besides morphometric characters mentioned above, all examined specimens of *R.antipai* including the NMNHS specimen can be clearly distinguished from *R.vladykovi* by the number of branched dorsal-fin rays, 8½, in contrast to 7½ found in all specimens of *R.vladykovi* examined in this study. Naseka (2001: 111) mentioned that 8½ rays can be rarely found in *R.vladykovi*; a revision of his primary data (radiographs) revealed a single specimen with 8½ branched dorsal-fin rays out of 46 examined. *Romanogobioantipai* further differs from *R.vladykovi* by the vertebral structure (Table 1, 2) having abdominal+caudal counts 19+21 or 20+21, which means that the caudal region is longer than the abdominal region vs. commonly (in 52 out of 70 specimens) 20+20 or 21+21 or variants with a caudal region shorter than the abdominal one.

**Figure 3.**
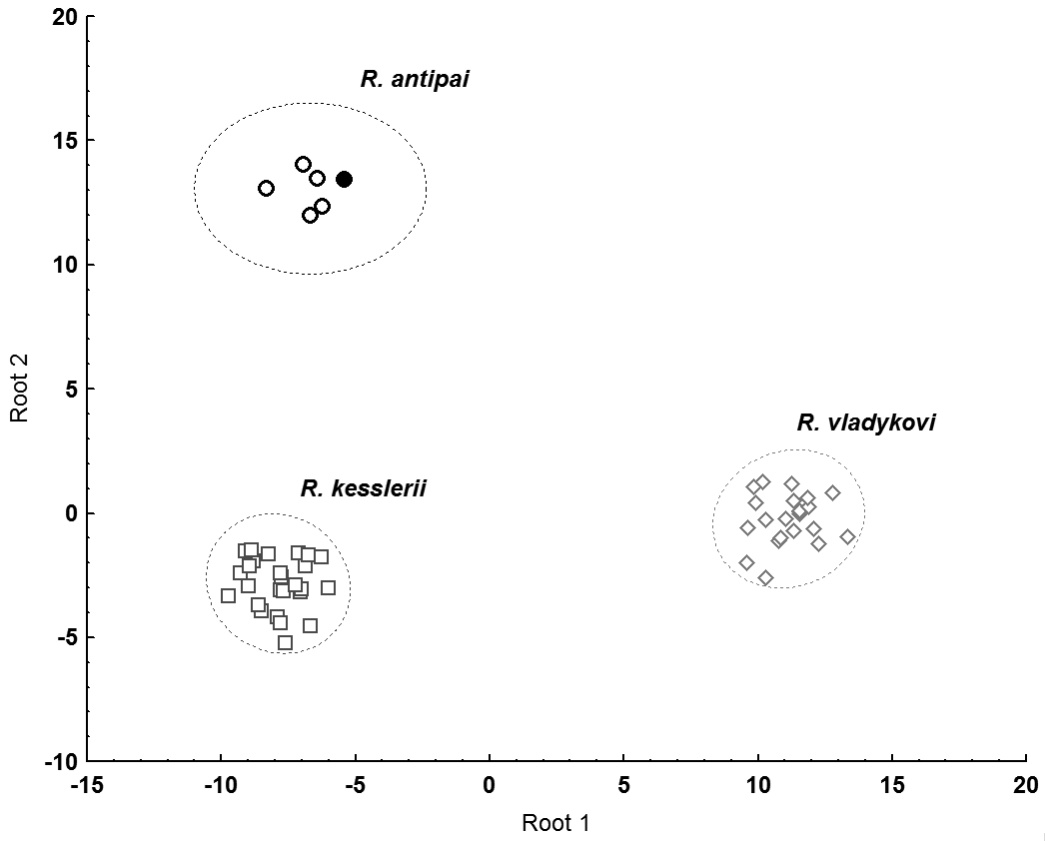
DFA (Euclidean distance, complete linkage clustering algorithm), distribution of discriminant scores along two canonical discriminant functions established to discriminate between three groups of samples (*R.antipai*, *R.kesslerii* and *R.vladykovi*). Solid circle corresponds to NMNHS specimen identified as *Romanogobioantipai*.

**Figure 4.**
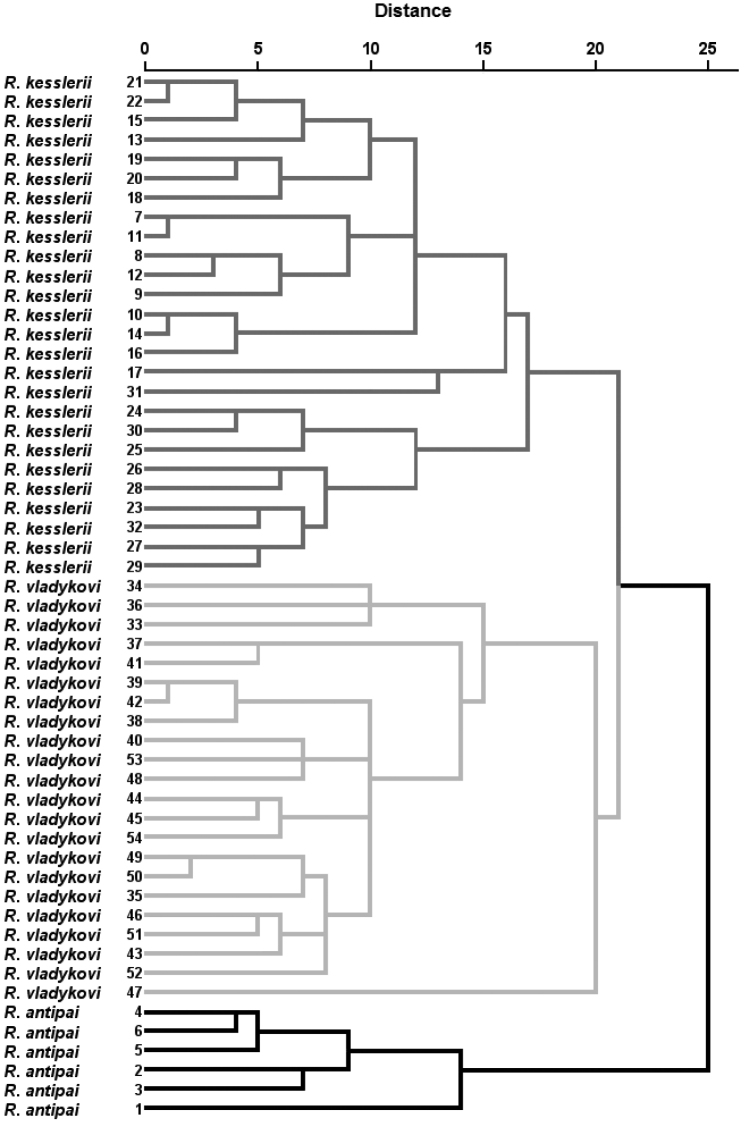
CA (SPSS, k-means) for three groups of samples (*R.antipai*, *R.kesslerii* and *R.vladykovi*). No 1 refers to NMNHS specimen identified as *Romanogobioantipai*.

A DFA (Fig. 3) showed differentiation of the three groups of samples identified as *R.antipai, R.kesslerii* and *R.vladykovi* (the number of unbranched dorsal-fin rays was excluded from the analysis as demonstrating zero variability within the groups) and the groups were 100% classified as predicted (Table 4). A CA (Fig. 4) supported the grouping.

**Table 4.** Results of DFA classification for three groups of samples (*R.antipai*, *R.kesslerii* and *R.vladykovi*).

Group	Classification matrix (*Romanogobio* 3 species)			
	Rows: Observed classifications			
	Columns: Predicted classifications			
	Percent correct	* R. antipai *	* R. kesslerii *	* R. vladykovii *
* R. antipai *	100.0	6	0	0
* R. kesslerii *	100.0	0	26	0
* R. vladykovii *	100.0	0	0	22
Total	100.0	6	26	22

To conclude, the analysis confirmed previously reported discriminating character states (number of branched dorsal-fin rays, relative size of the eye and the interorbital space, relative depth of the caudal peduncle) and introduces a new character (vertebral counts) for discriminating *Romanogobioantipai* from *R.kesslerii* and *R.vladykovi*. However, relative taxonomic status of these three species still waits for a phylogenetic analysis based on molecular data. It cannot be excluded that *R.antipai* is a deep-water ecophenotype of either *R.kesslerii* or *R.vladykovi*. The new record demonstrates that *R.antipai* is still extant in the lower Danube but at present can only be found at a greater depth in the main channel and the deltaic branches. Currently classified as Extinct using IUCN criteria, the conservation status of *Romanogobioantipai* needs revision, in light of the new record from 2016.
